# The Role of Genetic Testing in the Differential Diagnosis of Pustular Dermatoses: A Pediatric Case of Generalized Pustular Psoriasis Associated with the IL36RN Variant

**DOI:** 10.3390/jcm15093413

**Published:** 2026-04-29

**Authors:** Maksymilian Markwitz, Paweł Głuszak, Anna Skorczyk-Werner, Natalia Welc, Aleksandra Dańczak-Pazdrowska, Aleksandra Wnuk-Kłosińska, Monika Bowszyc-Dmochowska, Marian Dmochowski, Anna Wiśniewska-Szymańska, Sandra Ważniewicz, Adriana Polańska

**Affiliations:** 1 Department of Dermatology, Poznan University of Medical Sciences, 61-701 Poznan, Poland; 2 Doctoral School, Poznan University of Medical Sciences, 61-701 Poznan, Poland; 3 Department of Medical Genetics, Poznan University of Medical Sciences, 61-701 Poznan, Poland; 4 Department of Dermatology and Venereology, Poznan University of Medical Sciences, 61-701 Poznan, Poland

**Keywords:** DITRA syndrome, IgA pemphigus, Sneddon–Wilkinson syndrome, acute generalized exanthemous pustulosis, spesolimab, *IL36RN* gene

## Abstract

General pustular psoriasis (GPP) is a rare, potentially life-threatening neutrophilic dermatosis. Pediatric cases are uncommon and often misdiagnosed due to overlapping clinical and histopathological features with other pustular dermatoses. We present a case of an 11-year-old boy, initially diagnosed with Sneddon–Wilkinson syndrome, who presented with disseminated pustular eruptions, with no response to antibiotics, dapsone, and glucocorticosteroids. In histopathology, we observed subcorneal neutrophilic pustules. Due to atypical features and poor treatment response, the patient underwent genetic testing, which revealed a homozygous IL36RN gene c.338C>T (p.Ser113Leu) pathogenic variant, which enabled a definitive diagnosis of GPP. Treatment with acitretin led to clinical improvement. Pediatric GPP poses diagnostic and treatment challenges. Genetic testing for IL36RN pathogenic variants may aid in the diagnosis, especially in atypical cases. The presence of the biallelic IL36RN pathogenic variant supports the diagnosis of DITRA (Deficiency of the IL-36 Receptor Antagonist, ORPHA:404546)—a monogenic autoinflammatory form of GPP.

## 1. Introduction

Generalized pustular psoriasis (GPP) is a rare, severe neutrophilic dermatosis classified as a subtype of psoriasis; however, it is significantly different from plaque psoriasis in both clinical presentation and underlying pathogenesis [1]. GPP is potentially life-threatening and is characterized by the acute onset of widespread, disseminated, sterile pustules on erythematous skin [2,3]. These eruptions may be accompanied by systemic symptoms, such as fever, malaise, and pain, with or without extracutaneous manifestations, including arthritis. The clinical course of GPP is typically intermittent or relapsing, with acute flares that can be triggered by various factors such as infections, psychological stress, or may arise spontaneously without an identifiable cause [4]. Reported mortality rates range from 2% to 16%, underscoring the severity of the disease [5,6,7]. While the mean age of onset is commonly reported to be between 40 and 59 years, multinational cohort studies have documented a broader age range, with a mean onset of 31.0 ± 19.7 years, and GPP is also present in the pediatric population [6,7,8]. The pathogenesis of GPP is complex and not yet fully elucidated.

Recent studies have identified pathogenic variants in *IL36RN*, *AP1S3*, and *CARD14* genes in a large subset of patients, highlighting a genetic predisposition and implicating dysregulation of the innate immune response, particularly the IL-36 signaling pathway, in disease development [8,9,10]. This is further supported by the demonstrated clinical efficacy of spesolimab, a monoclonal antibody targeting the IL-36 receptor, in the treatment of GPP [11].

Diagnosis of GPP is primarily clinical, supported by histopathological examination [1]. Histologically, the condition is characterized by neutrophilic subcorneal pustules, with spongiform pustules of Kogoj described as the hallmark feature. However, diagnosis may be challenging, particularly in patients presenting at an unusual age with atypical clinical features, or in cases where prior systemic treatments, such as glucocorticosteroids, have altered the presentation. The differential diagnosis includes other pustular dermatoses, such as Sneddon–Wilkinson disease (known also as subcorneal pustular dermatosis, SPD, ORPHA: 293173), acute generalized exanthematous pustulosis (AGEP, ORPHA: 293173), and IgA pemphigus (ORPHA: 555905) [12,13].

We present the case of an 11-year-old boy who was initially diagnosed and treated as Sneddon–Wilkinson disease. This case also underscores the significance of genetic testing in the differential diagnosis of pustular dermatoses, particularly in challenging cases or those with atypical presentations.

## 2. Case Report

An 11-year-old boy was admitted to the Department of Dermatology with a referral diagnosis of Sneddon–Wilkinson disease for further diagnostics and treatment. Medical history included autoimmune thyroiditis, status post hypospadias repair, and being overweight. No medication allergies were reported. The patient was vaccinated according to the Polish national vaccination schedule.

**Figure 1:**
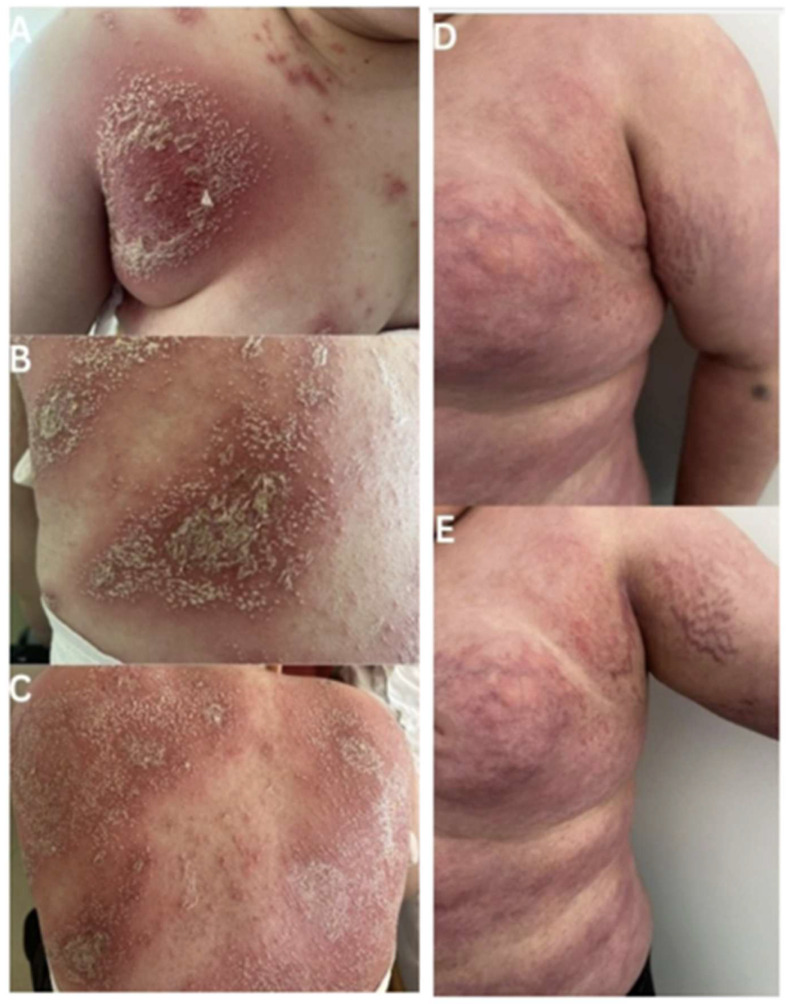
Clinical presentation of the patient. Initial flare-up characterized by disseminated pustules on erythematous skin, localized in the axillary area (**A**) and on the back (**B**,**C**). Presentation after initial treatment in the pediatric ward, on admission to the dermatology department. Visible signs include striae, obesity, skin atrophy, and teleangiectasia, all of which are consequences of prolonged glucocorticosteroid overuse (**D**,**E**).

The initial skin lesions, mild in severity, had appeared approximately one year prior and presented as papules and pustules in the axillary regions and scalp, which spontaneously regressed. Eight months after the onset of these initial symptoms, the patient developed erythematous lesions on the abdomen and back, disseminated pustules on the volar surfaces, and erythema with a whitish exudate in the perianal region. These lesions did not respond to treatment with topical and oral antibiotics. As a result, the patient was hospitalized in a pediatric ward. The initial clinical picture is shown in Figure 1. During this hospitalization, he developed a fever, elevated inflammatory markers, and was diagnosed with pneumonia. The diagnosis of pneumonia was established based on chest radiography. No microbiological investigations were performed, including culture or swab analysis of pustular lesions, and no additional microbiological evaluation was undertaken. At that stage, the cutaneous manifestations were initially considered to represent a pneumonia-associated exanthem. Thus, treatment including high-flow passive oxygen therapy, intravenous clindamycin, intravenous meropenem, intravenous immunoglobulin, and methylprednisolone at a dose of 1 mg/kg body weight was initiated. Despite this treatment, no significant improvement in dermatological condition was observed. Accordingly, an underlying dermatologic disorder was suspected, and a consultation with the Department of Dermatology was requested. Extensive diagnostic workup excluded HIV infection, lupus erythematosus, celiac disease, antiphospholipid syndrome, autoimmune blistering diseases, and other immune disorders.

**Figure 2:**
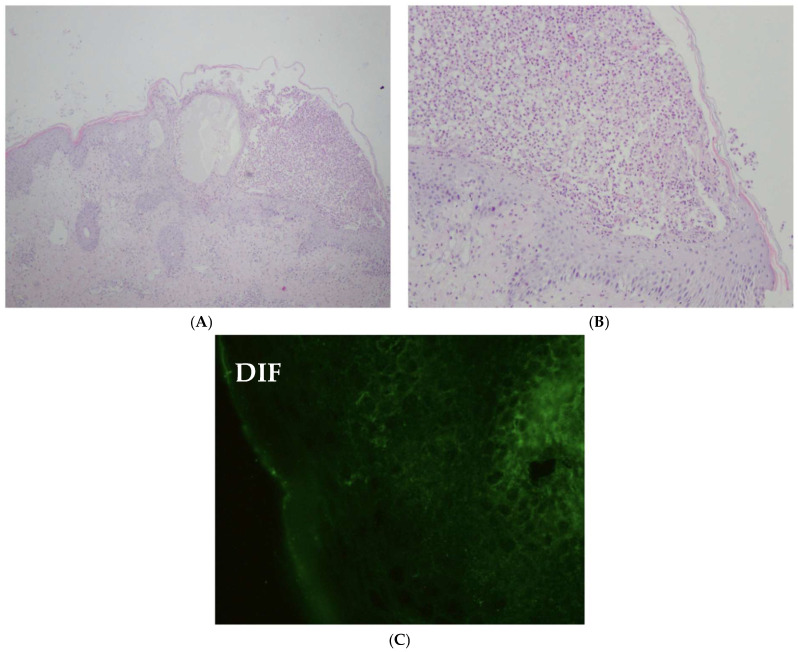
Histopathological findings (H&E staining) from a pustular lesion (**A**,**B**). In the epidermis, subcorneal neutrophilic pustules and vesicles containing neutrophils and occasional acantholytic cells are observed. In the dermis, there are moderate, perivascular, mixed inflammatory infiltrates, attenuated by edema. The findings may be consistent with Sneddon–Wilkinson disease, IgA pemphigus of the SPD type, AGEP, or GPP. Direct immunofluorescence (DIF) findings (**C**)—no IgA deposition was detected, ruling out both types of IgA pemphigus.

Histopathological examination revealed subcorneal neutrophilic pustules, vesicles containing neutrophils, and single acantholytic cells. These histopathological features are not disease-specific and may be observed in several pustular dermatoses, including generalized pustular psoriasis, Sneddon–Wilkinson disease, the subcorneal pustular dermatosis (SPD) variant of IgA pemphigus, and acute generalized exanthematous pustulosis. The absence of spongiform pustules of Kogoj rendered a diagnosis of GPP less likely. Direct immunofluorescence studies were negative, thereby excluding the SPD variant of IgA pemphigus from the differential diagnosis. Furthermore, the lack of an eosinophil-rich inflammatory infiltrate and absence of prominent papillary dermal edema argued against a diagnosis of AGEP. Based on these findings, a diagnosis of Sneddon–Wilkinson disease was initially made. Histopathological and immunopathological findings are shown in Figure 2. Treatment with dapsone 50 mg daily was initiated and well tolerated, resulting in initial clinical improvement. Methylprednisolone 2 mg daily was continued concurrently. After two months, due to rapid recurrence of symptoms, the dose of dapsone was increased to 75 mg daily and methylprednisolone to 8 mg daily. Two weeks later, dapsone had to be discontinued due to the development of hemolytic anemia requiring hospitalization and transfusion of one unit of red blood cell concentrate. At the same time, the dose of methylprednisolone was increased to 20 mg daily. Given the hemolytic anemia, glucose-6-phosphate dehydrogenase (G6PD) deficiency was suspected. G6PD activity was tested and found at the lower limit of normal (0.5 ng/mL; reference range 0.5–4.0 ng/mL). However, this assessment was performed after the hemolytic episode, which may lead to falsely normal results due to reticulocytosis and selective survival of G6PD-sufficient erythrocytes. Importantly, dapsone is known to induce oxidative hemolysis even in patients without confirmed G6PD deficiency, particularly in a dose-dependent manner. This underscores the need for careful hematologic monitoring during therapy, especially in pediatric patients.

On admission to the Department of Dermatology, the examination revealed widespread erythema on the trunk and limbs, telangiectasias, striae, and excessive adipose tissue, all consistent with steroid-induced changes. Scaling of the scalp, lower extremities, and hands was observed. The patient had geographic tongue; the oral mucosa was otherwise unremarkable. General physical examination revealed no significant abnormalities. During hospitalization in the Department of Dermatology, due to numerous steroid-related complications, a gradual reduction in methylprednisolone dose from 20 mg to 16 mg daily was initiated. On the following day, a few new pustules appeared in the axillary regions. The patient was started on acitretin 25 mg daily and discharged for outpatient follow-up. At follow-up visits after 7 and 21 days, significant clinical improvement was noted, with no active pustules remaining. Acitretin treatment was continued, and methylprednisolone was gradually tapered to 12 mg and then 8 mg daily.

**Figure 3:**
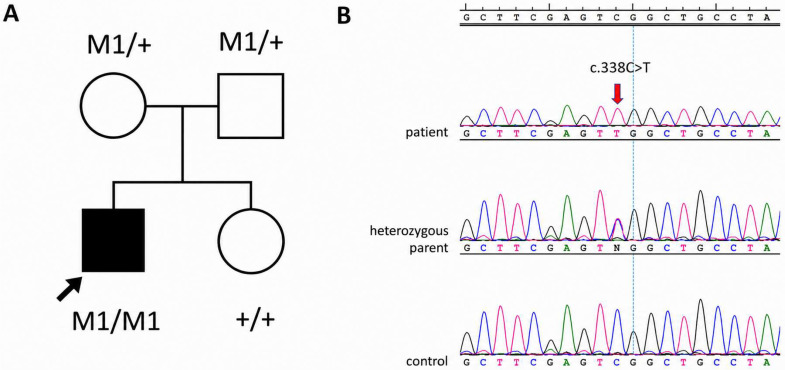
Pedigree of the family together with the segregation analysis results (**A**). The proband is marked with an arrow and a black-filled square. Unfilled symbols indicate unaffected individuals. M1: variant c.338C>T, +: wild type sequence. Chromatogram showing the c.338C>T p.(Ser113Leu) variant identified in the patient (**B**). The red arrow indicates the nucleotide that has been changed.

The presented case demonstrated an atypical clinical course, characterized by early disease onset and refractoriness to conventional therapeutic modalities, including dapsone and systemic glucocorticosteroids (GCS). Notably, attempts to taper GCS therapy consistently resulted in rapid recurrence of pustular flares. These findings prompted reconsideration of the initial diagnosis of Sneddon–Wilkinson disease. The persistence of recurrent pustular eruptions, along with the development of treatment-related adverse effects (including GCS-induced obesity and striae), further supported the need for diagnostic reassessment. Consequently, during hospitalization in the Department of Dermatology, an expanded diagnostic workup was undertaken, including targeted genetic testing for monogenic pustular psoriasis. Given the combination of early disease onset, recurrent sterile pustular flares, systemic inflammation, poor response to conventional therapies, and the absence of a clear drug trigger, IL36RN was prioritized as the initial molecular target. The patient was HLA-Cw6-negative. Genetic testing for pathogenic variants in the *IL36RN* gene, comprising exons 2-5 (exon 1 being non-coding), was amplified with PCR and directly sequenced. Sanger sequencing revealed a homozygous c.338C>T p. (Ser113Leu) pathogenic variant in exon 5. PCR conditions and primer sequences are available upon request. The results of segregation analysis were consistent with the expected autosomal recessive model of inheritance, confirming that both parents were heterozygous carriers of the pathogenic variant. The results of the segregation analysis, together with a pedigree of the family, are shown in Figure 3.

Based on the clinical presentation, histopathology, and genetic findings, the diagnosis was revised to GPP associated with the pathogenic variant in the *IL36RN* gene. Moreover, the patient fulfilled the criteria of DITRA syndrome (Deficiency of the IL-36 Receptor Antagonist)—a monogenic autoinflammatory variant of GPP. Given the revised diagnosis of GPP, corticosteroid tapering was continued, and acitretin treatment was maintained. The patient was eligible for future treatment with spesolimab upon reaching age 12 in case of future disease flares.

## 3. Discussion

Pediatric GPP remains a rare and diagnostically challenging entity, particularly due to its overlapping clinical features with other pustular dermatoses such as Sneddon–Wilkinson disease or autoimmune blistering diseases [1,12,13]. The rarity of pediatric cases, combined with atypical presentations and the absence of prior psoriasis vulgaris in many children, often delays and complicates the diagnosis. This case highlights the need for increased clinical suspicion and the incorporation of genetic testing to differentiate GPP from other pustular disorders, particularly in pediatric patients.

Accurate diagnosis is essential to ensure appropriate treatment. Sterile pustular eruptions may be manifestations of various distinct dermatoses, including generalized pustular psoriasis (GPP), subcorneal pustular dermatosis (SPD), acute generalized exanthematous pustulosis (AGEP), and IgA pemphigus [14,15,16,17,18,19]. Although some of these conditions present with characteristic clinical patterns: GPP often occurring in individuals with a history of psoriasis vulgaris, and AGEP typically being drug-induced, clinical distribution can also help in differentiation [14,19,20]. For example, SPD frequently presents as chronic, recurrent pustules localized to flexural areas [17,21].

Despite these clinical clues, additional diagnostic tests are often necessary. Histopathological examination and direct immunofluorescence (DIF) studies are particularly valuable in excluding autoimmune blistering diseases such as IgA pemphigus [21,22]. However, histopathological findings may sometimes be nonspecific or misleading. Subtle histological features, such as the presence of acantholysis, spongiosis, and eosinophilic infiltrates, can provide critical diagnostic insights when interpreted in the appropriate clinical context [15,16,17,18,19,20]. A detailed comparison of these aforementioned dermatoses, including their clinical, histopathological, and immunopathological characteristics, is presented in Table 1.

In cases where the diagnosis is unclear, histopathological findings are inconclusive, the disease presents at an early age, or there is resistance to standard treatment, molecular testing for specific pathogenic variants may be considered as an adjunct diagnostic tool. The most prevalent and well-characterized genetic pathogenic variants in GPP involve IL36RN, CARD14, and AP1S3, which primarily contribute to dysregulation of the IL-36 signaling pathway and enhanced NF-κB activation, driving the neutrophilic inflammation typical for this disease [23]. Pathogenic variants in several other genes, including *MPO*, *SERPINA1*, *SERPINA3*, *BTN3A3*, and *TGFBR2,* are less prevalent.

In the present case, *IL36RN* was prioritized for targeted testing because the patient presented with early-onset, recurrent, treatment-refractory generalized pustulation with systemic inflammation and no clear drug trigger, a clinical constellation strongly suggestive of an IL-36-driven pustular psoriasis spectrum disorder, particularly DITRA. In addition, the absence of prominent eosinophilic infiltrates or papillary dermal edema made AGEP less likely, while the atypical age at onset argued against classical SPD or IgA pemphigus. For these reasons, targeted Sanger sequencing of *IL36RN* was considered the most pragmatic first-line molecular approach. In similar cases, however, a broader multigene pustular psoriasis panel including *IL36RN*, *CARD14*, *AP1S3*, and, where available, other genes implicated in pustular/autoinflammatory phenotypes (e.g., *MPO*, *SERPINA1*, *SERPINA3*) may be preferable, particularly when the phenotype is mixed, family history is suggestive, or initial targeted testing is negative. Nevertheless, in comparable cases, we would advocate comprehensive sequencing of the relevant genes within a dedicated panel, where available, to better characterize the underlying genetic pathogenesis of the disease. Although recurrent hotspot variants have been identified in some genes, further studies are required to establish robust genotype–phenotype correlations.

IL-36 cytokine belongs to the IL-1 family [24]. IL36RN is a small gene encompassing six exons located on chromosome 2q14. The IL36RN gene product, IL-36Ra, inhibits the pro-inflammatory effects of IL-36 by binding to its receptor. This prevents the activation of downstream signaling pathways and reduces the production of pro-inflammatory cytokines, including IL-6, IL-8, IL-1α, and IL-36γ [24]. Multiple studies have demonstrated that IL36RN pathogenic variants are linked to GPP and other psoriasis subtypes, such as Acrodermatitis Hallopeau (AH) and palmoplantar psoriasis (PPP) [8,9,10,25,26]. A study by Twelves et al. showed that the most prevalent genetic pathogenic variants in GPP involve IL36RN (found in approximately 19% of patients) and CARD14, as well as AP1S3 (with allele frequencies of 3–5%). IL36RN pathogenic variants are significantly less common in PPP (3%) compared to GPP (19%) and ACH (16%) [8]. Another study by Krueger et al. demonstrated that IL36RN pathogenic variants occur significantly more often in GPP-only patients (without concurrent plaque psoriasis, with monoallelic pathogenic variants found in up to 33.3% and biallelic pathogenic variants in up to 73.2%, compared to frequencies of 0–11.9% and 0%, respectively, in plaque psoriasis alone [27]. IL36RN pathogenic variants are inherited in an autosomal recessive pattern. Patients carrying biallelic pathogenic variants tend to have worse disease manifestations, and the age of onset is lower [5,28]. The IL36RN p.Ser113Leu pathogenic variant, which was found in our patient, has been previously identified and is well-documented in the literature as the most common variant in the European population [10]. However, studies have also reported its presence in patients of non-European descent [9,10]. Due to the limited number of reported cases, no definitive genotype–phenotype correlations can currently be established for this pathogenic variant. Studies suggest that IL36RN pathogenic variants lead to atypical, treatment-resistant or pediatric presentations of GPP. In contrast to plaque psoriasis, where HLA-Cw6 is a well-established risk allele, its prevalence in patients with GPP ranges from 0% to 28.6%, aligning more closely with rates observed in the general population and suggesting a limited role in GPP pathogenesis [8,27].

DITRA syndrome (Deficiency of the IL-36 Receptor Antagonist) is the clinical manifestation of biallelic pathogenic variants in IL36RN [29,30]. It causes a monogenic autoinflammatory form of GPP. It was first described by Marrakchi et al. in 2011 [29]. It is characterized by recurrent GPP flares with systemic inflammation, often triggered by infections or stress, and is typically resistant to standard anti-psoriatic therapies [29]. The homozygous IL36RN c.338C>T (p.Ser113Leu) variant identified in our patient is the most frequent European DITRA variant. Given the early onset, recurrent pustular flares, lack of plaque psoriasis, and biallelic IL36RN mutation, this case fulfills the diagnostic criteria for DITRA. It is relevant, as it provides information about the prognosis and allows for proper genetic counseling.

Interestingly, similar genetic backgrounds have also been reported only for AGEP [14]. Cases of *IL36RN* pathogenic variants, previously described in GPP patients, have been identified in AGEP patients [31,32]. Moreover, IL-36γ is overexpressed in AGEP lesional skin, indicating its role in both GPP and AGEP. A 2019 study demonstrated that amoxicillin and letrozole can trigger IL-36γ production via TLR4 in CD14+ macrophages and keratinocytes in AGEP-positive patients, subsequently inducing IL-8 secretion in an IL-36-dependent manner [33]. There have also been cases of AGEP patients positive for *CARD14* gene pathogenic variants [34,35]. Moreover, cases of overlapping GPP and AGEP in patients with heterozygous *IL36RN* pathogenic variants are also described [36,37].

Currently, no pathogenic variants have been identified in patients with SPD, IgA pemphigus. Thus, while molecular testing may aid in the diagnosis of neutrophilic and pustular dermatoses, differentiation between GPP and AGEP cannot rely solely on pathogenic variant status; in such cases, a comprehensive medical history, alongside laboratory and histopathological evaluation, remains essential. This is particularly important in pediatric patients, in whom frequent exposure to systemic antibiotics may itself act as a trigger for AGEP, thereby further complicating the diagnostic assessment.

Therapeutically, management of pediatric GPP remains difficult due to the lack of robust evidence-based guidelines. While systemic corticosteroids and dapsone were initially administered in our case under the assumption of SPD, these treatments proved ineffective. This reflects broader observations from the literature, where non-retinoid first-line therapies often fail in pediatric GPP [7]. Moreover, prior corticosteroid or immunosuppressive treatment might blur the clinical picture and hinder diagnostic clarity. In suspected GPP, prolonged systemic GCS therapy should generally be avoided, particularly in children, because it may result in substantial toxicity and disease rebound during tapering. In selected severe cases with marked systemic involvement, short-term GCS may be considered as bridging therapy. This approach requires careful planning, close monitoring, and prompt quick transition to safer steroid-sparing agents. In our patient, prolonged GCS exposure was associated with striae, telangiectasias, skin atrophy, and excessive adipose tissue, while tapering was followed by the reappearance of pustules. Although this single case does not establish causality, it highlights an important clinical lesson: delayed recognition of GPP may expose pediatric patients to avoidable steroid-related toxicities and morbidity and postpone initiation of more appropriate therapy.

Retinoids, such as acitretin, remain the cornerstone of first-line therapy despite their slow onset of action and side-effect profile. According to the literature, in pediatric GPP, the most frequently used immunosuppressants are cyclosporine, retinoids, methotrexate and systemic GCS [7,38]. Although TNF-α inhibitors like infliximab, etanercept, and adalimumab have shown efficacy in adult GPP, their role in pediatric GPP remains poorly defined, due to limited data available [38,39,40]. Given the potential risks of long-term immunosuppression and secondary malignancies, current expert recommendations suggest TNF-a inhibitors should be reserved for severe, refractory cases or acute crisis intervention. New targeted therapies such as spesolimab, an IL-36 receptor antagonist, offer promising future options but are not yet broadly approved in the pediatric population [11]. Determining *IL36RN* pathogenic variant status in patients with GPP offers not only diagnostic insights but may also help predict treatment response. In the clinical trial of spesolimab, patients with *IL36RN* pathogenic variants achieved higher rates of complete pustule clearance (87.5% vs. 16.7%) and clear/almost clear skin (75.0% vs. 16.7%) at week one compared to placebo. Among patients without *IL36RN* pathogenic variants, clearance rates were lower but still favored spesolimab (42.9% vs. 0% for pustule clearance; 28.6% vs. 9.1% for clear/almost clear skin [41]. Regardless of pathogenic variant status, spesolimab provided rapid and sustained clinical improvement with a comparable safety profile [41]. In clinical practice, however, the use of spesolimab in pediatric patients remains constrained by age-related approval status, drug availability, and reimbursement policies, which vary across regions. Although our patient may become eligible for spesolimab upon reaching 12 years of age, treatment decisions in pediatric GPP must be individualized and based not only on molecular findings but also on disease severity, clinical course, and access to therapy. Identification of pathogenic variants in IL36RN may support consideration of IL-36-directed treatment, particularly in severe or refractory cases, as it provides a strong biological rationale for targeting this pathway. Nevertheless, genetic confirmation alone should not be regarded as a prerequisite for treatment initiation, nor does it fully predict therapeutic response. In real-world settings, optimal management requires a multidisciplinary approach involving dermatologists, pediatricians, and, when appropriate, geneticists, to balance efficacy, safety, and accessibility of available therapies.

Proper genetic counseling is essential in such cases. Our patient was found to have a biallelic *IL36RN* pathogenic variant, with both asymptomatic parents each carrying one mutated allele, while the patient’s sibling tested negative. This pattern indicates a 25% risk of recurrence for future offspring of the parents to inherit biallelic pathogenic variants and potentially manifest symptoms. Furthermore, the patient has a 100% probability of transmitting a mutated allele to his offspring. Given the incomplete understanding of phenotype-genotype correlations, it is possible that monoallelic pathogenic variants in his children could also lead to clinical manifestations.

## 4. Conclusions

This case highlights the diagnostic challenges of pustular dermatoses in pediatric patients, particularly when prior treatments blur the clinical picture. GPP should be considered in the differential diagnosis of recalcitrant pustular eruptions, even in atypical age groups and despite initial histopathological findings suggestive of other neutrophilic dermatoses such as Sneddon–Wilkinson disease. Molecular testing for *IL36RN* pathogenic variants plays a critical role in confirming the diagnosis of GPP as well as its spectrum (DITRA), guiding treatment decisions, and avoiding unnecessary or potentially harmful therapies. Early recognition of monogenic forms of GPP enables appropriate use of targeted treatments, such as IL-36 inhibitors, which can significantly improve patient outcomes. This case underscores the importance of integrating clinical assessment, histopathology, and genetic testing in the diagnostic algorithm for pustular dermatoses, particularly in children and in atypical presentations.

## 5. Future Directions

In daily clinical practice, artificial intelligence is increasingly being used as a tool to support the diagnostic process, particularly through the analysis of dermatological images. Recent studies have demonstrated that deep learning models and large language models with image recognition capabilities show high accuracy in the classification of skin lesions and in differential diagnosis [42,43]. Generalized pustular psoriasis in children may closely mimic other pustular dermatoses, both clinically and histopathologically. In the present case, this led to an initial diagnosis of Sneddon–Wilkinson disease and the initiation of treatment that proved ineffective. In such scenarios, image-recognizing artificial intelligence could help identify patterns of diffuse sterile pustulation, erythema, and treatment resistance that are disproportionate to more common diagnoses, thereby prompting reconsideration of the initial diagnosis.

In the future, integration of artificial intelligence into clinical practice may enable earlier detection of atypical inflammatory phenotypes and facilitate more accurate and timely diagnostic pathways. This could contribute to a reduction in time to correct diagnosis, as well as optimization of diagnostic and treatment costs. Such an approach may be particularly valuable in rare and heterogeneous conditions, such as pediatric GPP, where early recognition and appropriate molecular testing are critical for optimal management.

## Figures and Tables

**Table 1:** Differential diagnosis of pustular dermatoses considered in this case, with emphasis on molecular testing. Abbreviations: GPP—generalized pustular psoriasis; SPD—subcorneal pustular dermatosis; AGEP—acute exanthematous pustulosis; DIF—direct immunofluorescence study.

Condition	Typical Age	Clinical Pattern	Key Systemic and Laboratory Features	Key Histopathology Clues	DIF	Genetic Associations
Generalized Pustular Psoriasis (GPP)	Adults and children; may occur with or without prior psoriasis	Acute, widespread sterile pustules on erythematous skin; recurrent flares; may be triggered by infections, pregnancy, or corticosteroid withdrawal in psoriasis vulgaris patients	Fever, malaise, leukocytosis; possible arthritis	Spongiform pustules of Kogoj; intraepidermal neutrophilic pustules; acanthosis and papillomatosis	Negative	Well documented variants in *IL36RN*, *CARD14*, *AP1S3* (support IL-36–driven disease but not required for diagnosis)
Sneddon–Wilkinson disease (or subcorneal pustular dermatosis, SPD)	Typically middle-aged to elderly women	Chronic, relapsing superficial pustules in flexural/intertriginous areas; usually no clear trigger	Generally absent or mild systemic symptoms	Subcorneal pustules with neutrophils, with or without eosinophils; minimal spongiosis; otherwise preserved epidermis	Usually negative	No consistent genetic association identified
Acute Generalized Exanthematous Pustulosis (AGEP)	Any age, more common in adults	Acute eruption of numerous small pustules, typically drug-induced (e.g., antibiotics); rapid onset and resolution after trigger withdrawal	Fever, marked leukocytosis with eosinophilia; short disease course	Subcorneal and/or intraepidermal pustules with papillary dermal edema and mixed infiltrate rich in eosinophils; occasional keratinocyte necrosis	Negative	Variants in *IL36RN* or *CARD14* reported in some cases; may overlap with GPP
IgA pemphigus	Middle-aged to elderly	Subacute, pruritic pustules on erythematous base, often in flexural areas	Typically no systemic involvement	Neutrophilic pustules (subcorneal in SPD type; intraepidermal with acantholysis in IEN type)	Intercellular IgA deposition in epidermis	No consistent genetic association identified

## Data Availability

The authors confirm that the data supporting the findings of this study are available within the article.
